# Cholesterol Embolization Syndrome Presenting with Multifocal Cerebral Infarction After Thoracic Endovascular Aortic Repair: A Case Report

**DOI:** 10.3390/jcm14186507

**Published:** 2025-09-16

**Authors:** Seung Woo Lee, In Sook Kang

**Affiliations:** Department of Internal Medicine, College of Medicine, Ewha Womans University Mokdong Hospital, Seoul 07985, Republic of Korea; leeseungwoo1986@gmail.com

**Keywords:** cholesterol embolization syndrome, thoracic endovascular aortic repair, shaggy aorta, cerebral infarction, acute kidney injury, livedo reticularis, blue toe syndrome

## Abstract

We report a rare case of cholesterol embolization syndrome (CES) presenting with multifocal cerebral infarctions following thoracic endovascular aortic repair (TEVAR). A 75-year-old male with a 6.1 cm thoracic aortic aneurysm and extensive atherosclerotic disease (“shaggy aorta”) underwent zone 2 TEVAR with left subclavian artery embolization after left carotid-subclavian bypass surgery. Postoperatively, the patient developed altered mental status with multifocal cerebral infarctions involving the left middle cerebral artery territory, bilateral cerebellar hemispheres, and pons. The characteristic findings included livedo reticularis, blue toe syndrome, leukocytosis (21,190/μL), elevated C-reactive protein (11.4 mg/dL), and acute kidney injury (creatinine 3.81 mg/dL). Despite supportive care, including continuous renal replacement therapy, the patient died of sudden cardiac arrest on hospital day 123. This case demonstrates the devastating potential of CES following TEVAR in patients with a shaggy aorta, emphasizing the importance of preoperative risk stratification and early recognition of the characteristic clinical triad of neurological symptoms, cutaneous lesions, and renal dysfunction.

## 1. Introduction

Cholesterol embolization syndrome (CES), also known as cholesterol thrombus embolization or aeroembolism, occurs when cholesterol crystals and other debris are dislodged from atherosclerotic plaques in large proximal arteries and embolize into smaller distal vessels, causing end-organ damage through vascular occlusion and an associated inflammatory response [1]. The incidence of clinically evident CES varies markedly depending on the population studied and diagnostic criteria used. Following cardiac catheterization, the largest prospective study of 1786 patients reported a CES incidence of 1.4% [2]. CES commonly arises after invasive arterial procedures, including cardiac catheterization, carotid endarterectomy or stenting, and various cardiac, aortic, and other vascular surgeries [3]. In some cases, CES leads to cerebral infarction (CES-CI), presenting with a wide range of neurological symptoms such as confusion, focal neurological deficits, and headache [4]. Since its first Food and Drug Administration approval in March 2005, thoracic endovascular aortic repair (TEVAR) has revolutionized the treatment of descending thoracic aortic pathologies [5]. Initially developed specifically for the exclusion of thoracic aortic aneurysms, TEVAR is now the preferred minimally invasive approach over open surgical repair owing to its lower morbidity and mortality rates [6,7]. However, TEVAR is associated with complications, and embolic events are among the most concerning sequelae. Recent meta-analyses reported stroke incidence rates ranging from 3.2% to 8% after TEVAR [8]. Reported stroke rates after TEVAR (3.2–8%) mainly reflect thromboembolic events [8]; the incidence of cholesterol embolization syndrome (CES)-related cerebral infarction has not been clearly defined in large series [9]. The risk of embolic complications is particularly elevated in patients with extensive atherosclerotic disease, commonly referred to as “shaggy aorta,” which is characterized by irregular, ulcerated atherosclerotic plaques with mobile thrombi protruding into the aortic lumen [10]. Manipulation of guidewires, catheters, and endografts within the diseased aortic arch during TEVAR can dislodge atherosclerotic debris, leading to cholesterol embolization in various end-organs [11]. CES commonly presents with a characteristic triad of cutaneous manifestations (livedo reticularis and blue toe syndrome), acute kidney injury, and neurological symptoms [2]. Here, we present a rare case of CES manifesting as multifocal cerebral infarctions and systemic embolic phenomena in a 75-year-old man who underwent zone 2 TEVAR with left subclavian artery embolization for a thoracic aortic aneurysm with a shaggy aorta.

## 2. Case Description

**Figure 1:**
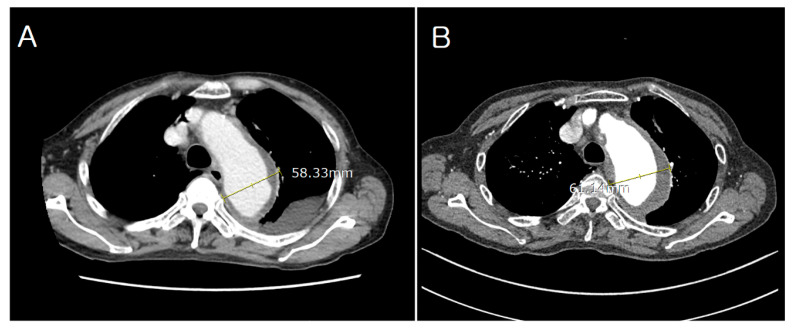
Preprocedural contrast-enhanced computed tomography (CT) of the thoracic aorta showing aneurysmal dilatation of the descending thoracic aorta. (**A**) Initial CT scan showing an aortic diameter of 5.8 cm. (**B**) Follow-up CT scan after 4 months showing progression to 6.1 cm.

**Figure 2:**
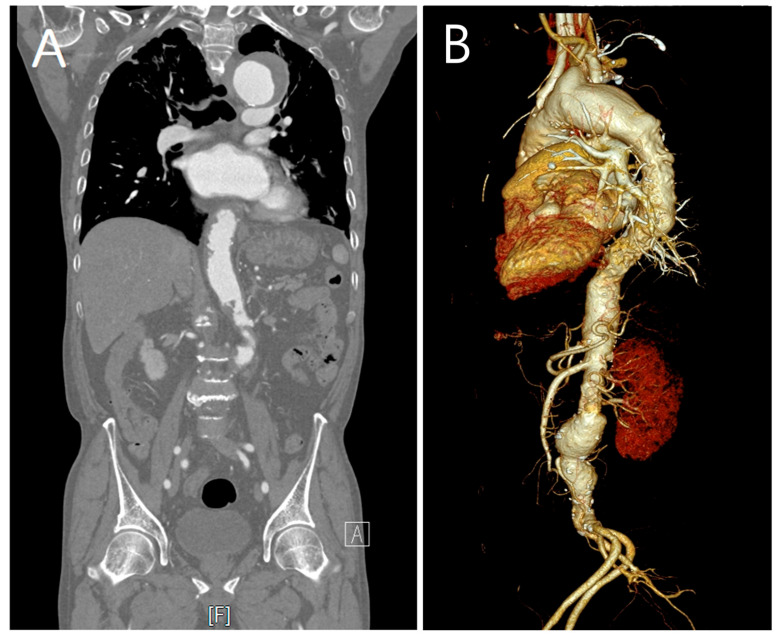
(**A**) Coronal contrast-enhanced computed tomography (CT) image showing atherosclerotic plaques along the entire aorta with diffuse luminal irregularity. (**B**) Volume rendering technique CT view showing extensive atheromatous changes and a “shaggy aorta” appearance, characterized by irregular intimal surfaces and calcified mural protrusions, indicating a high risk of cholesterol embolization. Labels: A = anterior; F = foot (orientation markers automatically generated by the CT system).

A 75-year-old male with a history of hypertension and emphysema presented to our hospital with dyspnea on exertion. Chest computed tomography (CT) revealed thrombosed aneurysmal dilatation of the aortic arch measuring approximately 5.8 cm in maximum diameter. Serial imaging demonstrated interval growth from 5.8 cm to 6.1 cm over 4 months, prompting the consideration of intervention (Figure 1). CT imaging also revealed extensive atherosclerotic plaques involving the entire aorta with diffuse luminal irregularity, consistent with a “shaggy aorta” appearance (Figure 2).

**Figure 3:**
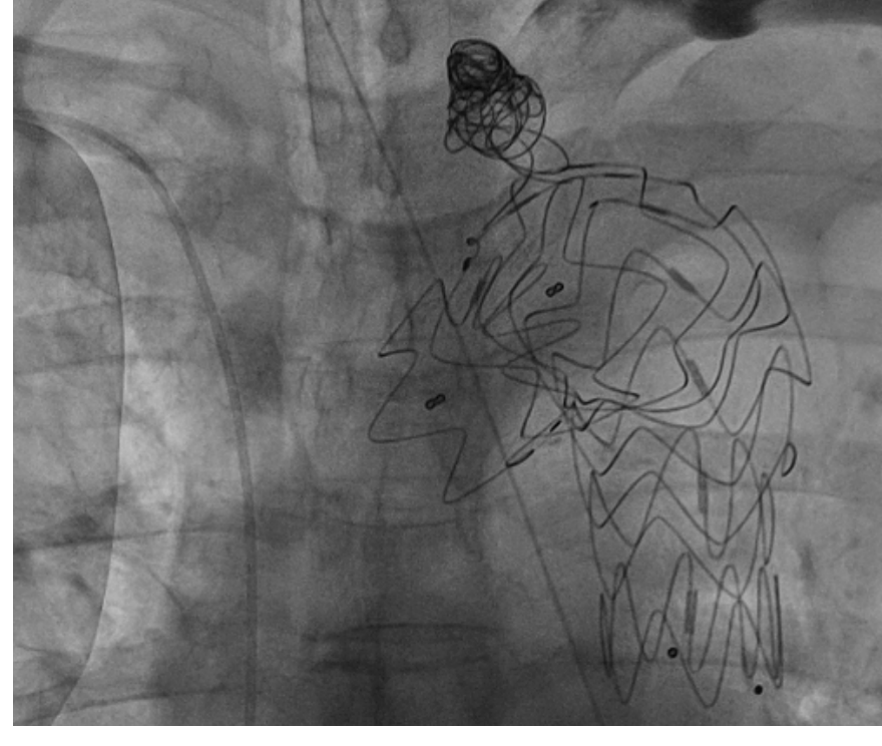
Fluoroscopic image showing successful deployment of the thoracic endograft in zone 2 of the aortic arch, along with coil embolization of the left subclavian artery to prevent type II endoleaks.

Given that the aneurysmal dilatation exceeded 5.5 cm in diameter and showed interval growth, TEVAR was planned. CT thoracoabdominal aortic angiography revealed preexisting left subclavian artery (LSCA) stenosis, and brain MRI showed hypoplastic stenosis in the right vertebral artery. After a multidisciplinary neurology consultation, it was determined that the risk of brain ischemia would be high if zone 2 TEVAR was performed without revascularization. Therefore, a left common carotid artery (LCCA)-to-LSCA bypass was planned before TEVAR. The patient was admitted for planned, staged procedures. During hospitalization, a newly detected junctional rhythm was identified, necessitating temporary pacemaker insertion via the left femoral vein. The cardiothoracic surgery team performed an LCCA-to-LSCA bypass under general anesthesia via a supraclavicular incision using an 8 mm InterGard graft (MAQUET Cardiovascular, Getinge, Gothenburg, Sweden). Two days after bypass surgery, TEVAR was performed under sedation with remifentanil. A Valiant 40 mm–36 mm, 150 mm stent graft (Medtronic Vascular, Santa Rosa, CA, USA) was successfully deployed in the distal portion of the LCCA under fluoroscopic and aortographic guidance using a marker pigtail catheter via the left common femoral artery. Following stent placement, coil embolization of the LSCA ostium was successfully performed using the left radial artery approach. The procedure was completed without immediate complications (Figure 3).

**Figure 4:**
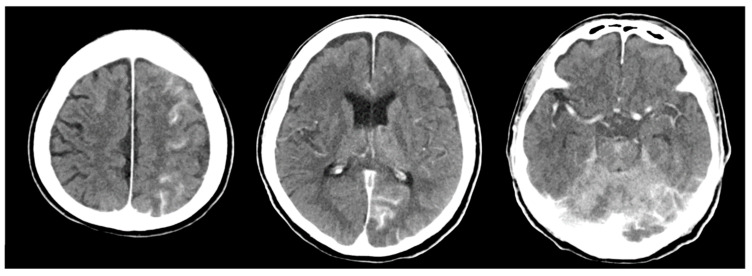
Brain CT showing multifocal infarctions in the left frontal and parietal lobes.

**Figure 5:**
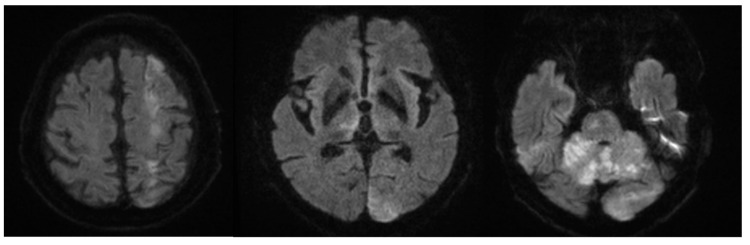
Diffusion-weighted MRI showing multifocal acute infarctions in the left middle cerebral artery border zone, bilateral cerebellar hemispheres, pons, and suspected involvement of both medial thalami.

The patient’s vital signs remained stable throughout the procedure. However, after discontinuation of remifentanil and transfer to the intensive care unit (ICU), the patient failed to regain full consciousness and remained in a stuporous state. Neurological examination revealed decreased motor strength in both the upper and lower extremities and leftward gaze deviation. Brain CT showed multifocal parenchymal enhancement within acute infarctions in the left frontal, parietal, and occipital lobes, as well as in the bilateral cerebellar hemispheres (Figure 4). Diffusion-weighted MRI confirmed multifocal acute infarctions in the left middle cerebral artery border zone, bilateral cerebellar hemispheres, pons, and the suspected bilateral medial thalami (Figure 5).

**Figure 6:**
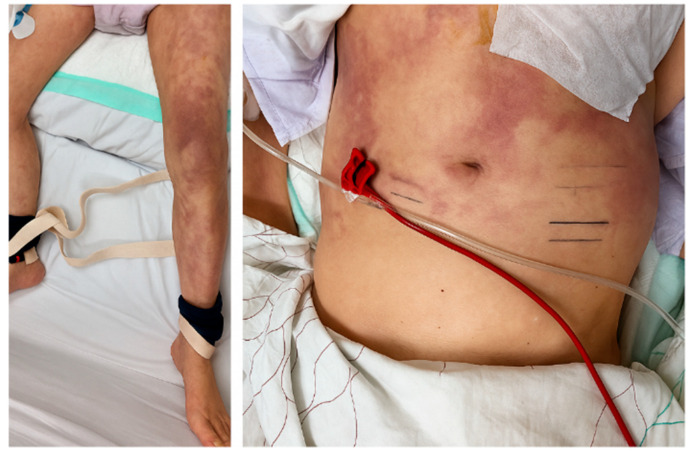
Livedo reticularis involving both lower extremities and violaceous mottling of the abdominal skin, consistent with cutaneous manifestations of cholesterol embolization syndrome.

**Figure 7:**
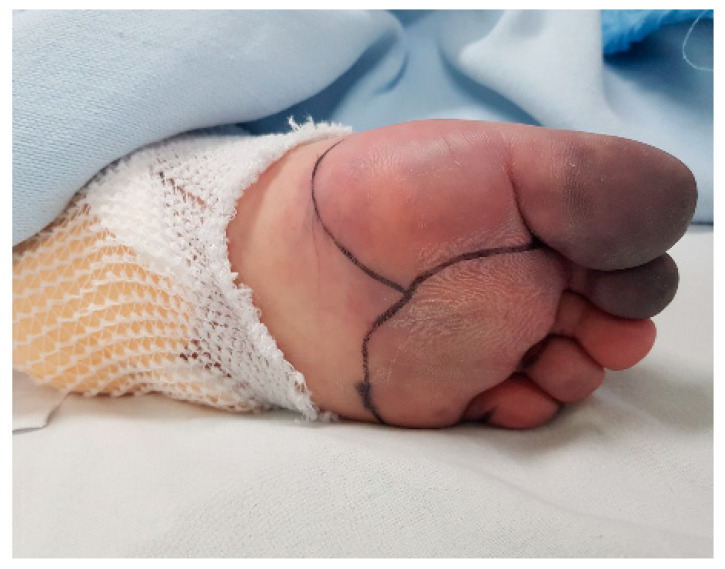
Bluish discoloration of the toes on both feet, characteristic of “blue toe syndrome,” a peripheral ischemic finding commonly associated with cholesterol embolization.

Physical examination revealed characteristic cutaneous manifestations, including livedo reticularis in both lower extremities and violaceous mottling of the abdominal skin (Figure 6). Bilateral bluish discoloration of the toes consistent with “blue toe syndrome” was also observed (Figure 7).

Laboratory evaluation revealed leukocytosis (21,190/μL), elevated C-reactive protein (11.4 mg/dL), and acute kidney injury, with serum creatinine increased to 3.81 mg/dL. Complement levels showed mildly decreased C3 (80.1 mg/dL) with normal C4 (26.8 mg/dL). The erythrocyte sedimentation rate increased to 23 mm/h, whereas the eosinophil count remained normal (Table 1).

Based on a constellation of clinical findings, multifocal brain infarctions following aortic manipulation, characteristic cutaneous lesions, acute kidney injury, and inflammatory markers, a diagnosis of CES was established. Treatment included the continuation of statin therapy, antiplatelet agents, and initiation of an angiotensin-converting enzyme inhibitor to prevent CES progression. Continuous renal replacement therapy (CRRT) was initiated for progressive renal dysfunction, and endotracheal intubation with mechanical ventilation was required owing to CO_2_ retention. External ventricular drainage (EVD) was performed to alleviate the progression of cerebral edema and the resulting increase in intracranial pressure owing to multiple brain lesions. Progressive necrosis in both toes was observed during the clinical course. Despite comprehensive supportive care in the ICU, including antibiotic treatment for pneumonia, CRRT, and mechanical ventilator support, the patient’s condition remained critical. Finally, the serum creatinine level stabilized, urine output improved, CRRT was discontinued, and the EVD was removed. The patient underwent a tracheostomy and was transferred to a home ventilator. His mental status gradually improved, and he was able to state his name and family members’ names and follow verbal commands. The patient was subsequently transferred to the general ward and referred to the rehabilitation department for continued therapy. Unfortunately, on hospital day 123, the patient experienced sudden cardiac arrest. After one episode of return of spontaneous circulation (ROSC), a second arrest occurred without recovery, and cardiopulmonary resuscitation was terminated. The patient was pronounced dead from sudden cardiac death.

## 3. Discussion

This case represents a rare but severe manifestation of CES after TEVAR in a patient with extensive atherosclerotic disease. The constellation of multifocal cerebral infarctions, characteristic cutaneous lesions, and acute kidney injury occurring immediately after aortic manipulation strongly supports the diagnosis of CES, highlighting the significant embolic risk associated with endovascular procedures in patients with “shaggy aorta.” Experimental studies have suggested that CES may represent an autoinflammatory condition in which inflammasome pathways involving NLRP3 and IL-1 are activated by cholesterol crystals [12]. The proposed pathophysiology involves both mechanical vascular occlusion and inflammatory responses, which can lead to progressive end-organ damage [1]. Cholesterol crystals have been shown to activate the IL-1β pathway via the NLRP3 inflammasome and to induce TNF and MIP2 secretion, mechanisms that may underlie the systemic inflammation observed in CES [12], although this was not directly demonstrated in our patient. The presence of “shaggy aorta” significantly elevates embolic risk during endovascular procedures. A novel scoring system assigns one point to each of the following: (1) ulcer-like thrombus, (2) maximum thrombus thickness ≥ 5 mm, and (3) mural thrombus occupying more than two-thirds of the aortic circumference [10]. Postoperative embolic complications were identified in 7.0% of patients who underwent TEVAR, with higher rates in patients with elevated shaggy scores [10]. The characteristic clinical presentation includes cutaneous manifestations (livedo reticularis and blue toe syndrome), acute kidney injury, and neurological symptoms ranging from confusion to focal deficits [2]. CES commonly leads to cerebral infarction with a wide range of neurological symptoms [4], as demonstrated in our case. Although peripheral eosinophilia is frequently observed in cholesterol embolization syndrome (approximately 20–80% of cases), it is not universally present and may be transient or delayed after the inciting event [13]. Therefore, a normal eosinophil count does not exclude CES. In our patient, the diagnosis was supported by the temporal association with aortic manipulation, characteristic cutaneous findings, multifocal cerebral infarctions, acute kidney injury, and elevated inflammatory markers. The gold standard for diagnosis is tissue biopsy, with kidney biopsy being diagnostic in >75% of cases [12]. Histopathological confirmation by renal or skin biopsy is considered the diagnostic gold standard [12]; however, this approach was not feasible in our frail patient due to acute kidney injury and progressive systemic illness. In such situations, clinical diagnosis is widely accepted in practice, particularly when supported by the characteristic triad of cutaneous manifestations, acute kidney injury, and neurological symptoms in temporal relation to aortic manipulation. While biopsy offers the highest diagnostic accuracy, clinical diagnostic criteria represent a reasonable and practical alternative in critically ill patients [12]. Stroke incidence rates after TEVAR range from 3.2% to 8% [8]. However, most studies do not distinguish between thromboembolism and cholesterol embolization; therefore, CES-related cerebral infarctions remain poorly characterized in the literature [9]. Although multifocal cerebral infarctions after TEVAR could also be explained by thromboembolic events from catheter manipulation, as has been described with catheter- or device-related dislodgement of atherosclerotic debris in diseased aortic arches [11], the absence of histological confirmation underscores the need for a balanced discussion of potential mechanisms. In our patient, however, the presence of a shaggy aorta, characteristic cutaneous manifestations, acute kidney injury, and elevated inflammatory markers in close temporal relation to the procedure strongly support systemic cholesterol embolization as the predominant cause. Recent studies have suggested the use of embolic protection devices as adjunct measures for stroke prevention during TEVAR. The Sentinel cerebral protection system has shown promising results in pilot studies; however, further research is needed [8]. Dual-filter cerebral embolic protection devices have demonstrated pathological confirmation of captured embolic debris [14]. Currently, no specific treatment exists for established CES, and its management remains primarily supportive. Colchicine, IL-1 inhibitors, and corticosteroids have been reported in small case series and observational studies as potential experimental approaches but their efficacy remains unproven and their role in routine management has not been established [12,15,16,17]. Colchicine blocks auto-inflammatory pathways, including NLRP3 and IL1 [15], and case reports have shown improvements with colchicine and corticosteroids [16]. High-dose corticosteroids have proven useful in the treatment of CES-associated renal failure [17]. In our patient, statins were continued for their lipid-lowering action as well as additional benefits including plaque stabilization, improvement of endothelial function, and anti-inflammatory effects [18]. Antiplatelet therapy was maintained in line with secondary prevention strategies for atherosclerotic disease [19]. An ACE inhibitor was initiated to control blood pressure and provide renal protection. Although direct evidence in CES is lacking, the use of ACE inhibitors is considered reasonable because of their proven cardiovascular benefits in patients with atherosclerotic disease [1]. Although immunosuppressive or targeted anti-inflammatory therapies have been reported in small series, these remain investigational [20] and were not pursued in our frail patient. Patients with multisystem CES have been reported to have poor prognoses, with mortality ranging from 58% to 90% and morbidities such as limb amputation or long-term dependence on renal replacement therapy [18]. Our case illustrates the devastating potential of this complication, despite aggressive supportive care. This case study emphasizes several key aspects. First, preoperative risk stratification using shaggy aorta scoring systems should guide procedural planning [10]. In our patient, retrospective application of the shaggy aorta scoring system [10] yielded a total score of 11, mainly driven by extensive mural thrombus ≥ 5 mm in thickness and occupying more than two-thirds of the aortic circumference in multiple segments. Although ulcer-like thrombus could not be definitively confirmed on axial images alone, this markedly elevated score far exceeds the proposed high-risk threshold of 3 [10] and is consistent with the patient’s clinical course and multiple embolic manifestations. Second, early recognition of the characteristic clinical triad of neurological symptoms, cutaneous lesions, and renal dysfunction is crucial for appropriate management [2]. Finally, the emerging understanding of CES as an autoinflammatory disease involving NLRP3 inflammasome activation opens up new therapeutic avenues warranting further investigation [12].

## 4. Conclusions

This case demonstrates the devastating potential of CES following TEVAR, despite appropriate procedural planning. This emphasizes the importance of careful risk stratification, early recognition, and the urgent need for effective preventive strategies and anti-inflammatory treatments in high-risk patients. Poor prognosis underscores the critical importance of patient selection and informed consent regarding embolic risk in patients with a shaggy aorta.

## Figures and Tables

**Table 1:** Laboratory findings on hospital day 1 and during clinical deterioration.

Lab	Hospital Day 1	During Clinical Deterioration	Reference
WBC	9.63	21.1	4.0~10.0 × 10^3^/µL
Eosinophils	4.2	0.2	0~5.8%
Hemoglobin	13.1	8.5	14.0~16.5 g/dL
Platelets	215	107	150~450 × 10^3^/µL
BUN	18	40	6~20 mg/dL
Creatinine	1.07	3.81	0.84~1.23 mg/dL
Estimated GFR	67	16	~≥90 mL/min/1.73 m^2^
Troponin	0.043	0.106	~0.014 ng/mL
CRP	1.01	11.4	~0.5 mg/dL
ESR	Not performed	23	0~20 mm/h
Serum cholesterol level	151	83	~200 mg/dL
C4 Complement	Not performed	26.8	10~40 mg/dL
C3 Complement	Not performed	80.1	90~180 mg/dL

WBC, white blood cell; BUN, blood urea nitrogen; GFR, glomerular filtration rate; CRP, C-reactive protein; ESR, erythrocyte sedimentation rate.

## Data Availability

The original contributions presented in this study are included in the article. Further inquiries can be directed to the corresponding author.

## Abbreviations

The following abbreviations are used in this manuscript:

CES	Cholesterol embolization syndrome
CI	Cerebral infarction
CRRT	Continuous renal replacement therapy
EVD	External ventricular drainage
ICU	Intensive care unit
LCCA	Left common carotid artery
LSCA	Left subclavian artery
ROSC	Return of spontaneous circulation
TEVAR	Thoracic endovascular aortic repair
